# Impact of age-specific immunity on the timing and burden of the next Zika virus outbreak

**DOI:** 10.1371/journal.pntd.0007978

**Published:** 2019-12-26

**Authors:** Michel J. Counotte, Christian L. Althaus, Nicola Low, Julien Riou

**Affiliations:** 1 Institute of Social and Preventive Medicine, University of Bern, Bern, Switzerland; 2 Graduate School for Cellular and Biomedical Sciences, University of Bern, Bern, Switzerland; Fundacao Oswaldo Cruz, BRAZIL

## Abstract

The 2015–2017 epidemics of Zika virus (ZIKV) in the Americas caused widespread infection, followed by protective immunity. The timing and burden of the next Zika virus outbreak remains unclear. We used an agent-based model to simulate the dynamics of age-specific immunity to ZIKV, and predict the future age-specific risk using data from Managua, Nicaragua. We also investigated the potential impact of a ZIKV vaccine. Assuming lifelong immunity, the risk of a ZIKV outbreak will remain low until 2035 and rise above 50% in 2047. The imbalance in age-specific immunity implies that people in the 15–29 age range will be at highest risk of infection during the next ZIKV outbreak, increasing the expected number of congenital abnormalities. ZIKV vaccine development and licensure are urgent to attain the maximum benefit in reducing the population-level risk of infection and the risk of adverse congenital outcomes. This urgency increases if immunity is not lifelong.

## Author summary

Zika virus (ZIKV) caused a major outbreak in the Americas between 2015–2017. It remains unclear if immunity after infection offers life-long protection at an individual level and how long herd immunity can protect a population against a new ZIKV outbreak. Data from Managua, Nicaragua showed an imbalance in protective immunity after ZIKV infection across different age-strata. We used this data to parameterize an individual based mathematical model to predict the future risk of a new ZIKV outbreak and to evaluate the effect of loss of immunity and the introduction of vaccination. We found that the 15–29 age range will be at highest risk of infection during the next ZIKV outbreak, increasing the expected number of congenital abnormalities. We show that vaccination could curb the risk of infection and could extend the herd immunity, but introduction within the next decade is crucial to provide the most benefit.

## Introduction

Zika virus (ZIKV) is a flavivirus, which is transmitted primarily by mosquitoes of the genus *Aedes*. Before 2007, circulation of the virus only occurred sporadically in African and Asian countries [1, 2]. Between 2007 and 2013, ZIKV caused large-scale epidemics in the populations of Micronesia [3], French Polynesia [4] and other Pacific islands [1]. ZIKV probably became established in *Aedes aegypti* mosquitoes in the Americas between 2013-2014, [5, 6] and then spread rapidly across the continent. In 2015, doctors in Brazil started reporting clusters of infants born with microcephaly, a severe congenital abnormality, and of adults with Guillain-Barré syndrome, a paralyzing neurological condition, resulting in the declaration by the World Health Organization (WHO) of a Public Health Emergency of International Concern (PHEIC) [7]. WHO stated, in September 2016, that ZIKV in pregnancy was the most likely cause of the clusters of microcephaly, and other adverse congenital outcomes [8, 9]. The risk of an affected pregnancy appears highest during the first trimester, with estimates between 1.0 and 4.5% [10, 11]. By the beginning of 2018, over 220,000 confirmed cases of ZIKV infection had been reported from Latin America and the Caribbean [12], which is estimated to be only 1.02% (± 0.93%) of the total number of cases, based on mathematical modelling studies [6].

Protective immunity conferred by infection, combined with high attack rates and herd immunity, can explain the ending of epidemics and the lack of early recurrence [13], as has been seen with ZIKV [14]. The duration of protective immunity induced by ZIKV infection remains uncertain, since immunity to ZIKV infection was not studied extensively before the 2013 outbreaks. Evidence from seroprevalence studies in French Polynesia and Fiji found that levels of ZIKV neutralizing antibodies decrease with time [15]. If the fall in antibody levels means that people become susceptible to infection again, population level ZIKV immunity might be declining already. Even if protective immunity is lifelong, the risk of a new ZIKV outbreak will rise as susceptible newborns replace older individuals, lowering the overall proportion of the population that is immune. A modelling study, based on data from the 2013 epidemic in French Polynesia, estimated that ZIKV outbreaks are unlikely to occur for 12 to 20 years, assuming lifelong immunity [16].

A direct consequence of population renewal will be an unequal distribution of immunity by age group, with younger age groups at higher risk from a new epidemic than older people [14]. That effect will be amplified if ZIKV attack rates are lower in children than adults. Assessing the risk of ZIKV infection in women of reproductive age is essential because ZIKV infection in pregnancy, leading to adverse congenital outcomes, has such important implications for individuals, for public health and for investment in surveillance and mitigation strategies, including vector control, early warning systems, and vaccines [17, 18]. However, no vaccine is currently available against ZIKV. Phase I clinical trials of ZIKV candidate vaccines have shown levels of neutralizing antibody titers that were considered protective against reinfection [19, 20]. Some vaccines have already entered phase II trials [21], but some companies have stopped vaccine development [22].

Researchers in Managua, Nicaragua were the first to report the age-stratified seroprevalence of ZIKV antibodies in population-based surveys [23]. The first cases of autochthonous ZIKV infection in Nicaragua were reported in January, 2016, and an epidemic was observed between July and December of that year. Through case-based surveillance, the public health authorities of Nicaragua reported a total of 2,795 people with ZIKV detected by reverse transcriptase (RT) PCR over this period [12]. The number of symptomatic infections is likely much higher, owing to under-reporting. Furthermore, ZIKV infection is asymptomatic in 33 to 87% of cases [23], which are generally not identified by surveillance systems. Shortly after the end of the 2016 epidemic, Zambrana et al. analyzed sera from two large population-based surveys in Managua to measure the prevalence of IgG antibodies against ZIKV in 2- to 14-year olds (N = 3,740) and 15- to 80-year olds (N = 2,147) [23]. The authors reported ZIKV seroprevalence of 36.1% (95% confidence interval, CI: 34.5; 37.8%) among the 2-14 year age group and 56.4% (95% CI: 53.1; 59.6%) among the 15-80 year age group [23, 24]. The observed post-outbreak seroprevalence in adults is in line with findings from seroprevalence studies from French Polynesia, Brazil, and Bolivia [25–27].

In this study, we used published data from the 2016 ZIKV epidemic in Managua and developed an agent-based model (ABM) to predict the evolution of age-specific protective immunity to ZIKV infection in the population of Managua, Nicaragua during the period 2017–2097. We assessed: 1) the risk of a future ZIKV outbreak; 2) the consequences of a future ZIKV outbreak on women of reproductive age; 3) the influence of loss of immunity on future attack rates; and 4) how vaccination could prevent future ZIKV outbreaks.

## Materials and methods

### Modelling strategy

We assessed the consequences of future outbreaks of ZIKV infection in Managua, Nicaragua using a stochastic ABM. The model follows a basic susceptible-infected-recovered (SIR) framework and integrates processes related to ZIKV transmission, immunity, demography, adverse congenital outcomes and vaccination (Table 1). We parameterized the model based on published estimates or inferences from data about the 2016 ZIKV epidemic (Table 1, Supporting information S1). We considered different scenarios about the duration of immunity, the timing and scale of ZIKV reintroductions in the population, and the timing and scale of a hypothetical vaccination program targeted towards 15 year old girls.

**Table 1:** Parameterization of the agent-based model.

Parameter	Comment	Source
**ZIKV epidemic parameters**		
Transmission rate^a^	Inferred from the 2016 epidemic^b^	[23]
Recovery rate	Inferred from the 2016 epidemic^b^	[23]
**ZIKV immunity**		
Initial immunity^a^	Inferred from the 2016 epidemic^b^	[23]
Duration of immunity	Lifelong or decaying with time	5 scenarios^c^
**Demography**		
Initial age distribution	–	[28]
Birth rate	–	[28]
Death rate^a^	–	[29]
Ageing	Linear ageing at each time-step	–
**ZIKV reintroduction**		
Delay until reintroduction	1 to 80 years	80 scenarios^c^
Cases reintroduced	1, 5 or 10 cases	3 scenarios^c^
**Risk of adverse congenital event**		
Exposure	Proportion of women in the first semester of pregnancy	[28]
Risk of microcephaly	Upon infection during exposure time (3 levels of risk)	[10, 11]
**Targeted vaccination**		
Date of implementation	In 2021, 2025 or 2031	3 scenarios^c^
Effective coverage	Proportion of 15 year old girls vaccinated (0% to 80%)	5 scenarios^c^

^*a*^age-dependent parameters;

^*b*^inferred from the 2016 epidemic by fitting a compartmental SIR model to these data, see Supporting information S1;

^*c*^the different scenarios are discussed in the text in detail under the headings corresponding to the headings of this table.

### Model structure

We simulated a population of 10,000 individuals for 80 years (2017–2097). We assigned agents’ age and ZIKV infection status (susceptible *S*, infected *I* or immune *R*). Initial conditions reflected the situation in Managua, Nicaragua in 2017, when there was no documentation of active transmission. In the outbreak-free period, we only considered demographic and immunity processes: births, deaths, ageing and, if applicable, loss of immunity and vaccination. Given the scarcity of these events at the individual level, we selected a long time-step of seven days and stochastically applied the transition probabilities at each time step for each agent. After a given time, ZIKV-infected cases were reintroduced in the population. Upon reintroduction, the time step was reduced to 0.1 days, and we evaluated the epidemic-related transition probabilities: Susceptible agents may become infected at a rate *β*_*a*_*I*/*N*, where *β*_*a*_ is the age-dependent transmission rate and N the total population size. Infected individuals may recover with a rate *γ*. We ignored the influence of the vector population and assumed that the force of infection is directly proportional to the overall proportion of infected individuals. We allowed six months for the outbreak to finish after introduction. Simulations were conducted independently for each combination of scenarios and repeated 1,000 times. In the baseline scenario, we assumed no vaccination, no loss of immunity and a reintroduction of 10 infected individuals.

We implemented the model in ‘Stan’ version 2.18 [30] and we conducted analyses with R version 3.5.1 [31]. The Bayesian inference framework Stan permits the use of probability distributions over parameters instead of single values, allowing for the direct propagation of uncertainty. Stan models are compiled in C++, which improves the efficiency of simulations. The algorithm in Supporting information S1 describes the ABM in pseudo code. The model code and data are available from http://github.com/ZikaProject/SeroProject.

### Parameterization

#### ZIKV epidemic parameters

We inferred the probability distributions for the age-specific transmission rate *β*_*a*_ and the recovery rate *γ* from data on the 2016 ZIKV epidemic in Managua, Nicaragua. We used surveillance data [23], which give weekly numbers of incident ZIKV infections, confirmed by RT-PCR (dataset *A*, n = 1,165), and survey data on age-stratified ZIKV seroprevalence, measured among participants of pediatric and household cohort studies in Managua during weeks 5–32 of 2017 (dataset *B*, n = 3,740 children and 1,074 adults) [23].

We conducted statistical inference using a deterministic, ordinary differential equation (ODE)-based version of the ABM with three compartments (*S*, *I* and *R*) and two age classes (*a* ∈ {1, 2} corresponding to ages 0–14 and ≥15):

$$\begin{array}{cc} \frac{dS_{a}}{dt} & =-\beta_{a}S_{a}\frac{\sum I_{a}}{N} \end{array}$$ (1)

$$\begin{array}{cc} \frac{dI_{a}}{dt} & =\beta_{a}S_{a}\frac{\sum I_{a}}{N}-\gamma I_{a} \end{array}$$ (2)

$$\begin{array}{cc} \frac{dR_{a}}{dt} & =\gamma I_{a} \end{array}$$ (3)

We ignored demography in this model because it covers a short time span. We recorded the overall cumulative incidence of ZIKV cases using a dummy compartment:

$$\begin{array}{c} \frac{dC}{dt}=\sum_{a}\beta_{a}S_{a}\frac{\sum I_{a}}{N} \end{array}$$ (4)

in order to compute the weekly incidence on week *t*:

$$\begin{array}{c} D_{t}=C\left(t\right)-C\left(t-1\right) \end{array}$$ (5)

We fitted the model to weekly incidence data *A* using a normal likelihood after a square-root variance-stabilizing transformation [32]:

$$\begin{array}{c} \mathrm{Pr}\left(A|\beta_{a},\gamma ,\rho ,\sigma \right)=\prod_{t}N\left(\sqrt{A}|\sqrt{\rho D},\sigma \right) \end{array}$$ (6)

where *ρ* is a reporting rate parameter and *σ* an error parameter. In addition, we also fitted the model to the number of individuals with anti-ZIKV antibodies at the end of the epidemic by age group *B*_*a*_ using a binomial likelihood:

$$\begin{array}{c} \mathrm{Pr}\left(B|\beta_{a},\gamma \right)=\prod_{a}B\left(B_{a}|n_{a},p_{a}\right) \end{array}$$ (7)

where *B*_*a*_ the number of individuals with antibodies, *n*_*a*_ is the sample size in each age group, and *p*_*a*_ = *R*_*a*_(*t*_*end*_)/*N*_*a*_(*t*_*end*_) the proportion of immune at the end of the epidemic. The full likelihood was obtained by multiplying Eqs 6 and 7. We chose weakly-informative priors for all parameters and fitted the model in Stan (Table 2). We describe the calculation of the basic reproduction number $R_{0}$ in Supporting information S1. We used one thousand posterior samples for *β*_*a*_ and *γ* obtained by Hamiltonian Monte Carlo in the ABM model, ensuring the propagation of uncertainty of these parameters. In Supporting information S1 we provide a schematic representation of the models and the information flow. Parameter values can translate from deterministic to agent-based versions of an epidemic model if the time step is small [33], which was the reason for using a time step of 0.1 days.

**Table 2:** Parameter estimates inferred from incidence and sero-prevalence data on the 2016 ZIKV epidemic in Managua, Nicaragua.

Parameter	Interpretation	Prior	Posterior (median and 95% CrI)
*β*_1_	Transmission for age group 0-14	Expon(0.1)	0.19 (0.16; 0.22)
*β*_2_	Transmission for age group ≥15	Expon(0.1)	0.32 (0.30; 0.36)
1/*γ*	Duration of infectious period	Gamma(1, 0.1)	4.8 (4.3; 5.4)
*ρ*	Reporting rate	Beta(1, 1)	0.24% (0.21; 0.26)
*I*(0)	Initial number of infectious	Expon(0.1)	74 (40; 134)
$R_{0}$	Basic reproduction number	–	1.58 (1.56; 1.59)

CrI: Credible interval.

#### ZIKV immunity

We used the deterministic model, described in the previous section, to infer the proportion of people with protective immunity within each age group at the end of the 2016 epidemic ${\tilde{p}}_{a}$. We used one thousand posterior samples of ${\tilde{p}}_{a}$ in the ABM to allow the propagation of uncertainty. Protective immunity to ZIKV after infection was lifelong in our first scenario, so the reduction of the overall proportion of immune individuals in the population decreased only because of population renewal. Given the absence of evidence about the duration of immunity to ZIKV, we considered four scenarios assuming exponentially distributed durations of immunity with means of 15, 30, 60, 90, or 150 years. These values correspond to a proportion of initially immune agents that loses immunity after 10 years of 55%, 28%, 15%, 11% or 6%, respectively (Supporting information S1).

#### Demography

We based the initial age distribution of the population on data from the World Bank [34]. We used age-dependent death rates for 2016 from the World Health Organization [29]. For births, we computed a rate based on an average birth rate in Nicaragua of 2.2 births per woman, which was uniformly distributed over the female reproductive lifespan [28]. We defined the period of reproductive age between 15 and 49 years. The ageing process was linear, increasing the age of each agent by 7 days at each 7-day time step.

#### ZIKV reintroduction

We reintroduced ZIKV in the population after a delay of *d* = {1, …, 80} years in independent simulations. We chose this approach rather than continuous reintroductions to remove some of the stochasticity and assess more clearly the association between immunity decay and risk of an outbreak. As the probability of an extinction of the outbreak depends on the number of ZIKV cases reintroduced in the population, we considered three different values for the seed (1, 5 or 10 cases) and compared the results (Supporting information S1). Simulations using continuous reintroductions each year are presented in the Supporting information S1.

#### Risk of adverse congenital outcomes

The estimated number of microcephaly cases resulting from the reintroduction of ZIKV depended on the exposure, i.e. the number of pregnant women infected by ZIKV during their first trimester, to which we applied three different levels of risk, based on published estimates [10, 11]. We obtained the number of ZIKV infections among women aged 15–49 years from ABM simulations. As gender was not explicitly considered in the model, we assumed that women represented 50% of the population. We assumed a uniform distribution of births during the reproductive period, and considered that the first trimester constituted a third of ongoing pregnancies at a given time. We explored three different levels of risk of microcephaly in births to pregnant woman with ZIKV infection during the first trimester, as reported by Zhang et al. (2017), based on data from French Polynesia (0.95%, called low risk) and Brazil (2.19% and 4.52%, called intermediate and high risk, respectively) [6, 10, 11].

#### Vaccination

We examined the effects of a potential ZIKV vaccine, given to 15-year-old-girls. This vaccination strategy was used for rubella virus, which also causes congenital abnormalities, before the vaccine was included in the measles, mumps and rubella vaccine given in early childhood [35]. The main objective of vaccination would be the prevention of adverse congenital outcomes, including microcephaly. We simulated this intervention in the ABM, assuming vaccine implementation starting in 2021, 2025 or 2031. From that date, half of the agents reaching age 15, representing females, could transition to immune status *R* regardless of their initial status, with an effective vaccination coverage ranging from 20% to 80%.

### Outcome analysis

From the simulations, we collected 1) the evolution of the age-specific ZIKV immunity in the population; 2) the attack rate resulting from the reintroduction of ZIKV at year d; 3) the age of newly infected individuals. We fitted a binary Gaussian mixture model to dichotomize the observed attack rates into either outbreaks or non-outbreaks. We defined the outbreak threshold as the 97.5% upper bound of the lower distribution. This corresponded to a threshold of 1%, so that attack rates ≥1% were considered as outbreaks. The age structure of newly infected individuals was used to compute relative risks of infection by age group.

### Sensitivity analysis

We explored the effect of seasonality, of changes in vector density, of migration, and of an endemic circulation of ZIKV on our predictions regarding the attack rate and the proportion of introductions that result in an outbreak. Different scenarios, methods and assumptions are provided in Supporting information S2.

## Results

### 2016 ZIKV epidemic

**Fig 1:**
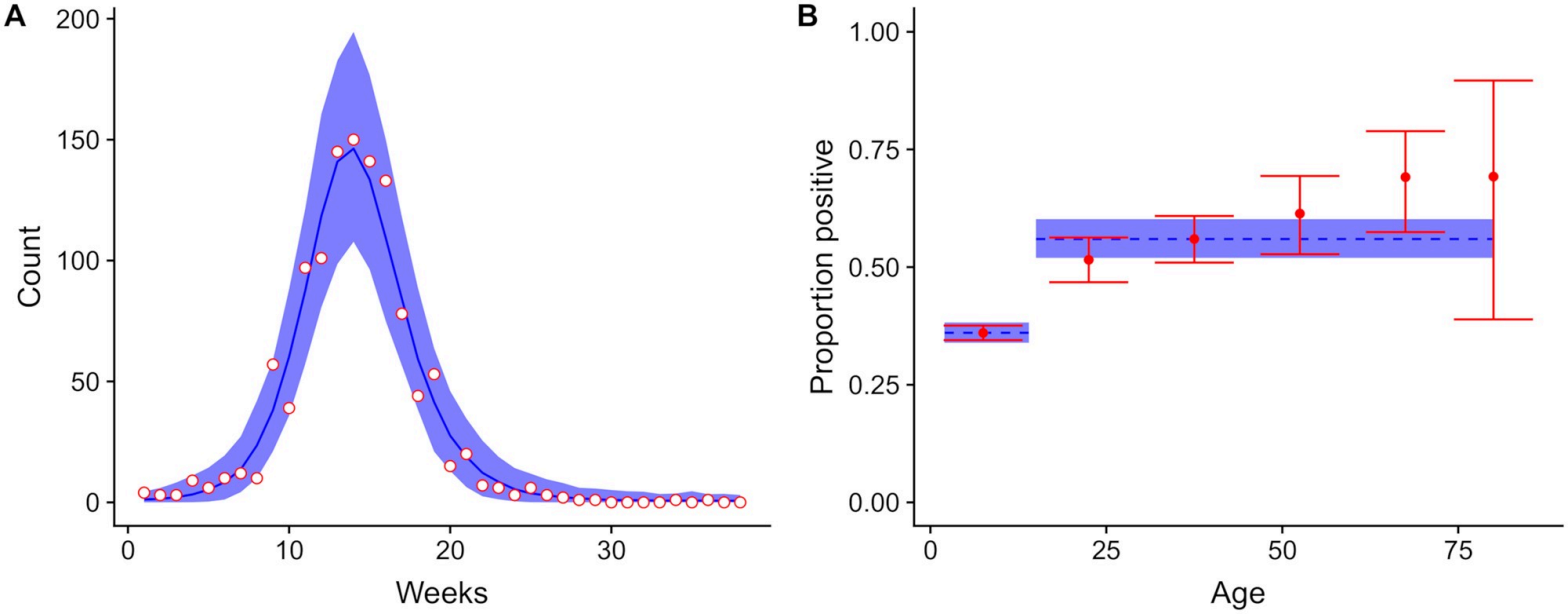
Model fit for the 2016 ZIKV epidemic. Model fit for (A) weekly incidence data and (B) post-epidemic sero-prevalence data from the 2016 ZIKV epidemic in Managua, Nicaragua. Data points are in red and the corresponding model fit (posterior median and 95% credible interval) is in blue.

The fitted model (Fig 1), resulted in a reporting rate of 0.24% (95% credible interval, CrI: 0.21; 0.26). The transmission rate in the 0-–14 age group was 42% (95% CrI: 35; 48) lower than in the ≥15 age group. This corresponded to an overall basic reproduction number $R_{0}$ of 1.58 (95% CrI: 1.56; 1.59). The predicted percentage of immune at the end of the epidemic was 36% (95% CrI: 34; 38) for the 0–14 age group and 53% (95% CrI: 50; 57) for the ≥15 age group.

### Immunity and population

**Fig 2:**
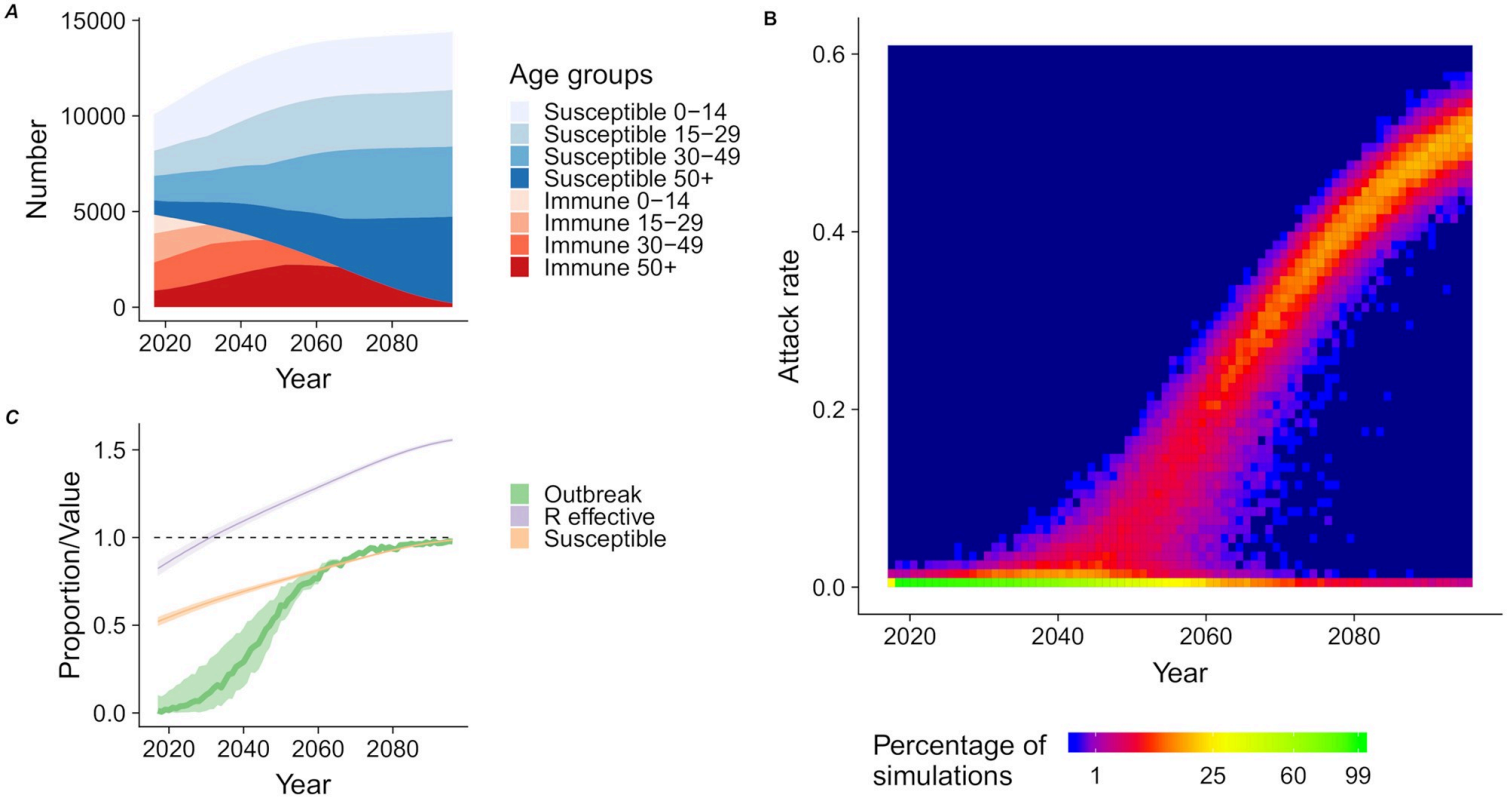
Future risk of ZIKV outbreaks. (A) The evolution of the immunity status per age group in a population of 10,000 agents for the next 80 years based on the demographic structure of Nicaragua. (B) Heat map of the distribution of the attack rates resulting from the reintroduction of ZIKV in the population at each year (1000 simulations for each year). (C) The evolution of the proportion of reintroductions resulting in outbreaks (with a threshold of 1%) with time (green), proportion of susceptible (orange), and effective reproduction number $R_{e}$ (purple).

In our forward simulations, the expected population size increased by 42% between 2017 and 2097. Under the assumption that ZIKV infection results in lifelong protective immunity, population renewal will create an imbalance in the proportion immune in different age groups. We expect the overall proportion of the population with protective immunity to have halved (from 48% to 24%) by 2051 and to be concentrated among the older age classes (Fig 2A). The 0–14 year old age group will become entirely susceptible by 2031 and the 15–29 year old age group by 2046.

### Future risk of ZIKV outbreak

Reintroductions of ZIKV in the population of Managua are unlikely to develop into sizeable outbreaks before 2035, 24 years after the 2016 epidemic, assuming lifelong immunity for individuals infected in 2016 (Fig 2B). After this point, attack rates resulting from ZIKV reintroduction will rise steeply. By 2047, we predict that ZIKV reintroductions will have a 50% probability of resulting in outbreaks with attack rates greater than 1% (Fig 2C). In 2047 the median attack rate of successful introductions is 3.6% (IQR: 2.0–6.2).

### Risk of infection and microcephaly births in women of reproductive age

**Fig 3:**
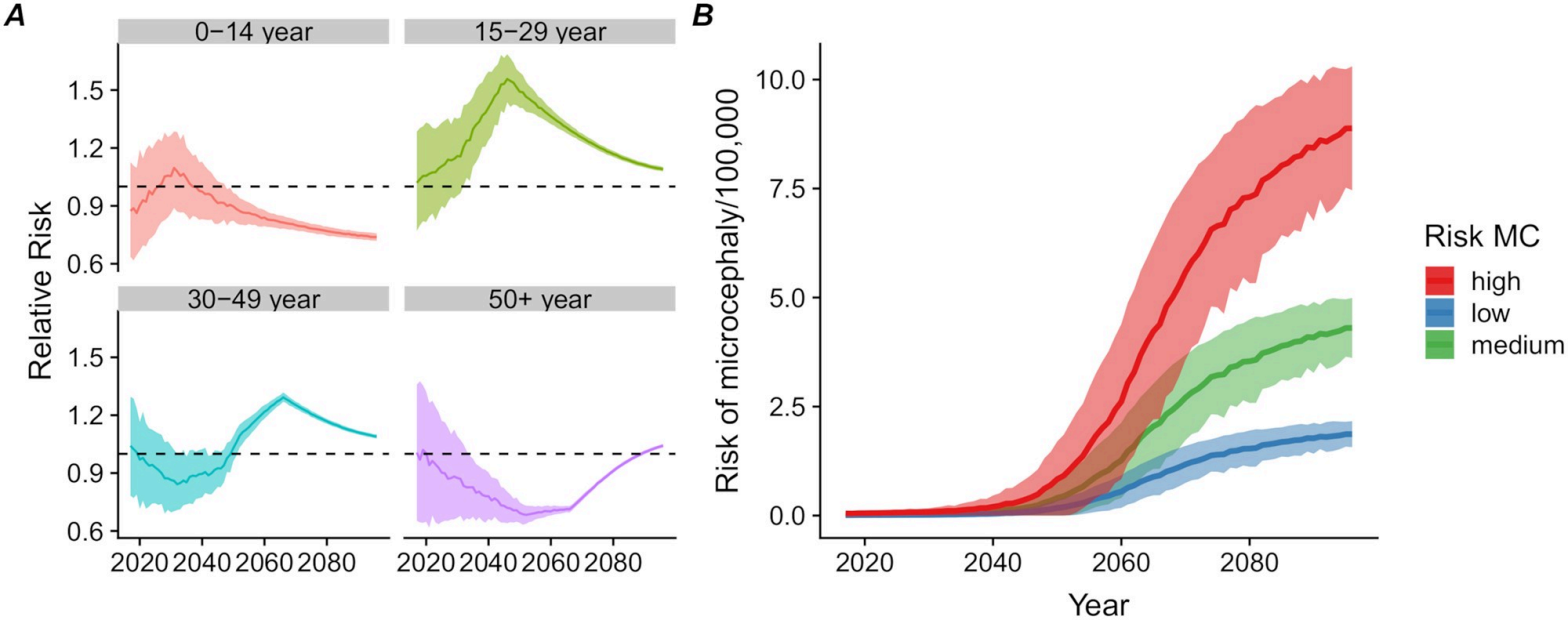
Risk of infection and microcephaly births in women of reproductive age. (A) Relative risk of ZIKV infection during a ZIKV outbreak per age group compared to the general population by year (median, interquartile range). (B) Expected number of additional microcephaly events associated with ZIKV infection during pregnancy per 100,000 total population according to three different risk scenarios.

The differences between age groups in both immunity and transmission will result in a disproportionate burden of infection in the 15–29 year age class. The relative risk of infection in this age group ranges from 1.2 to 1.6, compared with the general population if an outbreak occurs during the period 2032–2075 (Fig 3A). As most pregnancies occur in this age group, these women are also the most likely to experience a pregnancy with an adverse outcome. The increased risk of infection in this group implies that the number of adverse congenital outcomes resulting from a ZIKV outbreak during this period is likely to be higher than expected with a homogeneous distribution of immunity across ages. Assuming different values for the added risk of microcephaly after a ZIKV infection during the first trimester, we expect the mean number of additional microcephaly cases due to ZIKV infection resulting from the reintroduction of the virus in Managua, Nicaragua to reach 1 to 5 cases per 100,000 population in 2060 (Fig 3B).

### Loss of immunity

**Fig 4:**
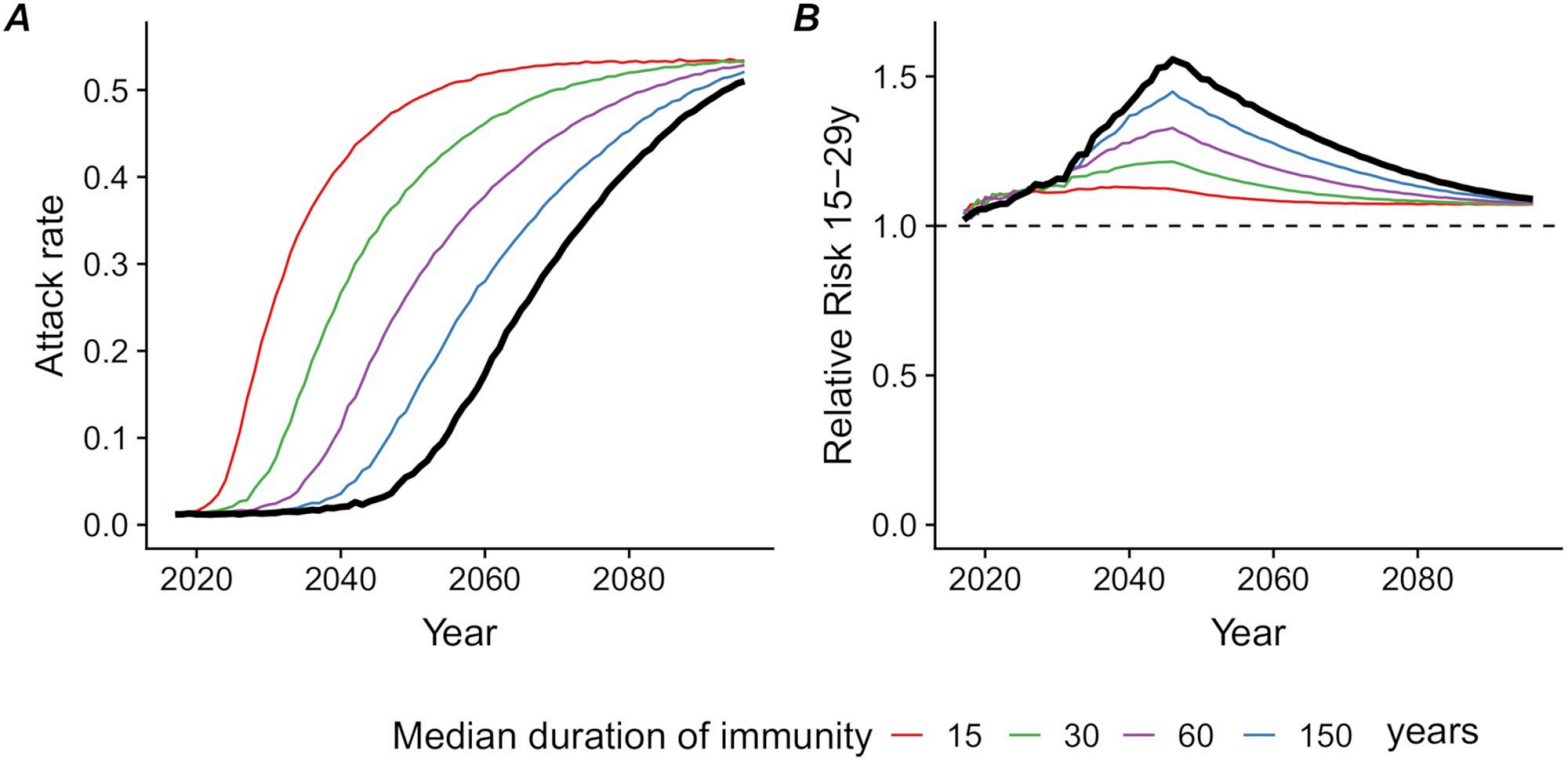
Loss of immunity. Consequences of alternative scenarios regarding the mean duration of protective immunity (15, 30, 60 and 150 years), compared with lifelong immunity (thick black line): (A) median attack rate of ZIKV among reintroductions resulting in outbreaks (with a threshold of 1%) and (B) relative risk of ZIKV infection during an outbreak in the 15–29 year age group compared with the general population.

If protective immunity to ZIKV is not lifelong, the time window before observing a rise in the attack rates resulting from ZIKV reintroduction will shorten (Fig 4A). For instance, if 15% of the those who were infected in 2016 lose their immunity after 10 years (a mean duration of immunity of 60 years), the time until the risk of outbreak upon reintroduction reaches 50% would be 14 years earlier (2033) than with lifelong immunity (2047). If 55% lose their immunity after 10 years (a mean duration of immunity of 15 years), in 2024, 50% of the introductions result in an outbreak, and the attack rate in 2047 is 47%. Loss of immunity over time would reduce the relative risk in the 15–29 year old age group (Fig 4B).

### Targeted vaccination

**Fig 5:**
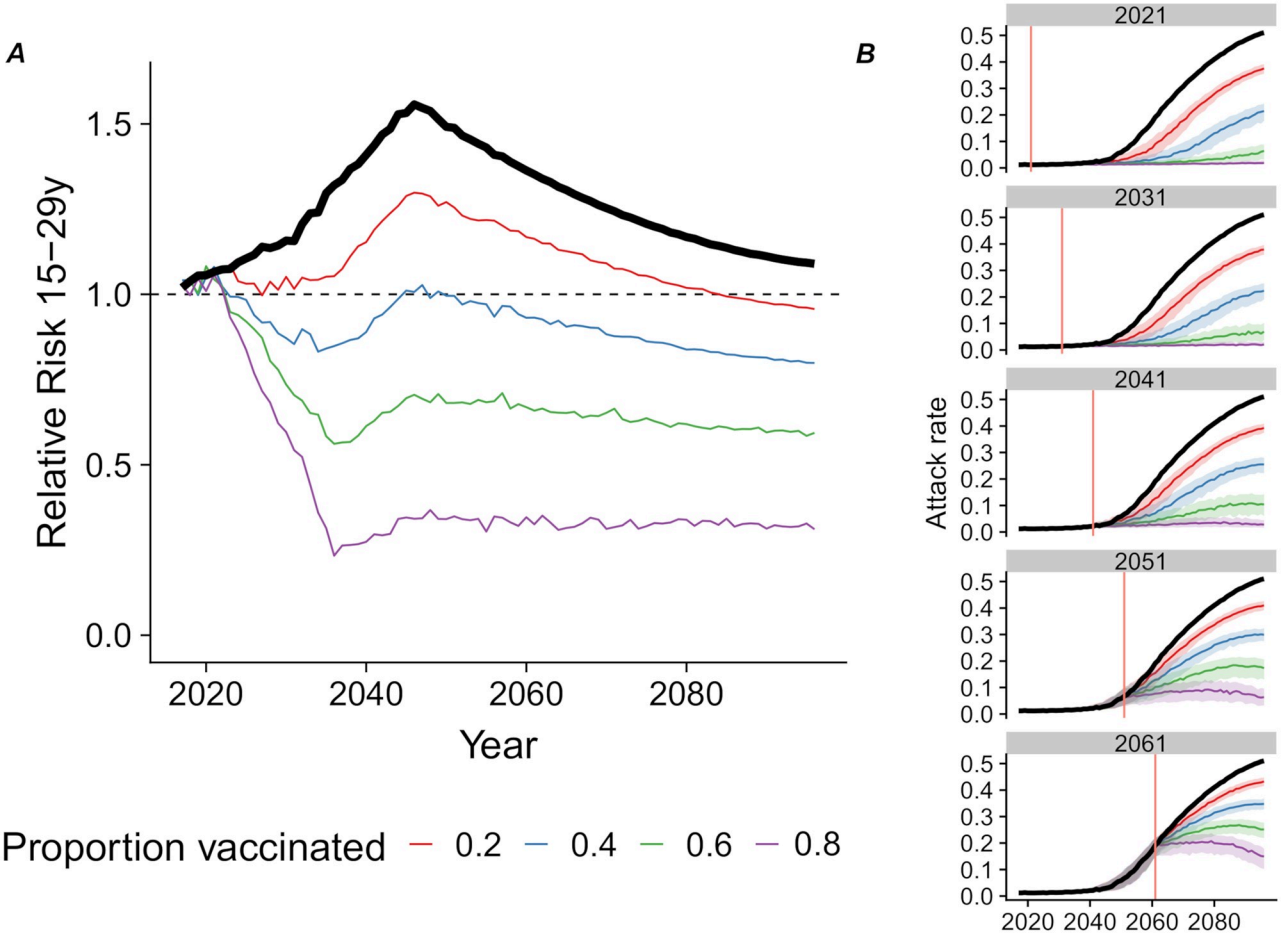
Targeted vaccination. Consequences of implementing a targeted vaccination program among 15-year-old-girls from 2021 onwards with various levels of effective vaccination coverage (from 20 to 80%) compared with no vaccination (thick black line). (A) relative risk of ZIKV infection during an outbreak in the 15–29 year age group compared with the general population and (B) attack rate of ZIKV among reintroductions resulting in outbreaks (median, interquartile range, with a threshold of 1%), when vaccination is introduced from 2021, 2031, 2041, 2051 or 2061 onwards (red vertical line).

The implementation of a vaccination program targeted towards 15 year old girls between 2021 and 2031 would reduce the risk of infection in women aged 15-29 years and would also indirectly reduce the overall risk of a ZIKV outbreak in the population (Fig 5). If effective vaccine coverage is 60–80% amongst 15 year old girls, the prolongation of herd immunity could effectively mitigate the overall risk of a ZIKV outbreak in the population. The reduction in the number of microcephaly cases would then exceed what would be expected by considering only the direct protection granted by a vaccine to future mothers. A later implementation of the intervention would be less effective, as it becomes more difficult to maintain the herd immunity (Fig 5B).

### Sensitivity analysis

We considered additional model features that may impact our predictions in a sensitivity analysis (Table 3 and Supporting information S2). Accounting for seasonality or for a future increase in vector abundance would result in higher transmission rates. This would lead to a shorter time window until a rise in the risk of ZIKV outbreak, and higher overall attack rates. A future diminution of vector abundance would have the opposite effects. Human migration from rural areas to Managua, Nicaragua would lead to a sharper decline of protective immunity in the population, also lowering the time window before the next ZIKV outbreak. Finally, a continuous endemic circulation of ZIKV in the region would increase the probability of an outbreak early on and lead to more stochasticity.

**Table 3:** Summary of the impact of features considered in the sensitivity analysis.

Scenario	Consequence on the model	Years until 50% of reintroductions result in an outbreak (Year)	Attack rate in 2047 (IQR)
Baseline	–	31 years (2047)	3.6% (2.0–6.2)
Seasonality	Increased transmission rate (+12%)	21 years (2037)	15.3% (11.2–18.4)
Increased vector abundance	Increased transmission rate (+12%)	21 years (2037)	15.3% (11.2–18.4)
Decreased vector abundance	Decreased transmission rate (–12%)	44 years (2050)	1.6% (1.3–2.5)
Migration	Influx of susceptible individuals	23 years (2039)	12.7% (8.7–16.2)
Endemicity	Continuous reintroductions	23 years (2039)	2.5% (1.7–4.1)

## Discussion

In this mathematical modelling study, we show that a new ZIKV outbreak in Nicaragua would affect proportionally more women in the young reproductive age range (15–29 years) than the general population, owing to the age-dependent infection pattern and population renewal. The risk of a new ZIKV outbreak in Nicaragua, after reintroduction, will remain low before 2035 because of herd immunity, then rise to 50% in 2047. If protective immunity to ZIKV decays with time, ZIKV recurrence could occur sooner. Timely introduction of targeted vaccination, focusing on females aged 15 years would both reduce the risk of adverse congenital outcomes and extend herd immunity, mitigating the overall risk of an outbreak and resulting in lower attack rates if an outbreak occurs.

### Strengths and limitations of the study

A strength of our approach is that it allows for the propagation of uncertainty from the initial data into the risk assessment, by transferring the posterior distributions of the parameters from the deterministic model fitted to surveillance and seroprevalence data on the 2016 epidemic into the ABM used for simulations. Roche et al. showed that, when a sufficiently small time step was chosen, stochastic and deterministic models using the same parameter values led to similar results [36]. Additionally, we benefited from the availability of high quality data from population-based surveys that included participants from age 2 to 80 years in Managua, Nicaragua. The age-stratified seroprevalence data allowed us to investigate the risk in different age groups and better assess the evolution of the age-specific immunity, which is crucial when studying adverse congenital events caused by ZIKV infection during pregnancy.

We chose a simple approach based on an SIR structure, similar to the model used by Netto et al. (2017), to focus on the dynamics of infection and immunity in the human population [26]. We did not model vector populations and behavior explicitly, as in some other studies [14, 16, 37]. This simplification limits the mechanistic interpretation of the epidemic parameters, but provides a phenomenological description of the transmission dynamics. We believe that this approach is appropriate because our main objective was to determine the risk of an outbreak after reintroduction of ZIKV, which is mostly influenced by the level of protective immunity in the human population. We acknowledge that the future occurrence of ZIKV in the area also depends on the presence of a competent vector. Our choice is supported by sensitivity analyses that show that more complex model structures (delayed SIR and Ross-MacDonald-type models) were not superior to a simple SIR structure in describing the 2016 ZIKV epidemic of Managua (Supporting information S1). Similarly, Pandey et al. (2013) showed that additional model complexity does not result in a better description of the dynamics of transmission of dengue virus (another Aedes-borne virus) in a human population compared with a SIR model [38]. In our model, the transmission rate (*β*_*a*_) captures both human-mosquito and mosquito-human transmission; we assumed a constant transmission rate, as observed in the 2016 outbreak.

Despite having modeled the effect of migration on our predictions, uncertainty remains; factors such as the political instability in Nicaragua could drive migration and influence disease transmission, as we currently observe in Venezuela and bordering countries [39].

### Interpretation in comparison with other studies

This study shows that the lower attack rate of ZIKV in children than in adults will hasten the emergence of a population that will be fully susceptible to infection, especially if immunity is not lifelong. The advantage of our approach is that we used the age-specific attack rates to model the processes of ageing in relation to protective immunity to ZIKV explicitly. Even with lifelong immunity, our model predicts that children aged 0–14 years will become entirely susceptible by 2031 and 15–29 year olds by 2046. In future outbreaks, the attack rate will then be highest amongst 15–29 year olds, including women who will be at risk of ZIKV infection in pregnancy. If immunity wanes, the time until the next ZIKV outbreak will be reduced and, in that case, the distribution of infection risk would be more equal across age groups (Fig 4). Several authors have studied the time to a next ZIKV outbreak, but none studied the effect of the loss of immunity over time in relation to age. Assuming lifelong immunity, our estimates of the time until the risk increases are similar to the 12–20 years before re-emergence estimated for French Polynesia [16]. Netto et al. (2017) used an SEIR model to show that in Salvador, Brazil, the effective reproduction number was insufficient to cause a new outbreak during the “subsequent years” [26]. Lourenço et al. (2017) showed the same for the whole of Brazil: herd immunity should protect the population from a new outbreak in the coming years [40]. Ferguson et al. (2016) concluded that the age distribution of future ZIKV outbreaks will likely differ and that a new large epidemic will be delayed for “at least a decade” [14].

Other ZIKV vaccination studies confirm our findings. However, they do not show the effect in risk groups nor assume herd immunity from previous outbreaks as we did; Durham et al. (2018) showed that immunizing females aged 9 to 49 years with a 75% effective vaccine and a coverage of 90%, would reduce the incidence of prenatal infections by at least 94% [41]. Similarly, Bartsch et al. (2018) showed that women of childbearing age or young adults would be an ideal target group for vaccination [42]. Valega-Mackenzie et al. (2018) formulated a vaccination model for ZIKV transmission that included mosquito and sexual transmission [43]. They found that vaccination works if high coverage is achieved, both when sexual transmission or vector-borne transmission is most important.

### Implications and future research

Our finding that people in the 15–29 year age range are more at risk of infection implies that we expect a higher number of congenital abnormalities due to ZIKV infection. Thus, vaccine development efforts should be increased. Our conclusions are drawn based on data from Managua, Nicaragua, but should be relevant to many regions in the Americas and the Pacific that have documented high post-epidemic levels of seropositivity [25–27]. In regions where ZIKV has not yet caused an epidemic but competent vectors are present, vaccination would be in place as well. Further age-stratified seroprevalence studies, using sensitive and specific tests and with longitudinal follow-up, are needed to improve our understanding of ZIKV antibody distribution in populations and to quantify the duration of immunity. This information will provide important information to improve mathematical modeling of ZIKV risk.

ZIKV vaccine development faces considerable hurdles. First, the evaluation of vaccine efficacy has stalled because the reduced circulation of ZIKV has reduced the visibility of ZIKV-associated disease [22]. Second, it remains unclear if neutralizing antibodies induced by vaccination are sufficient to protect women against vertical transmission and congenital abnormalities [44]. Third, it is not clear whether or how vaccine-induced antibodies against ZIKV will cross-react with other flaviviruses. To move vaccine development forward, we need to find regions where disease will occur to be able to conduct trials. This requires identifying populations that are at risk, and implementing surveillance there. These can either be regions where ZIKV is endemic, or where ZIKV outbreaks are likely to occur; throughout the Americas, there might be regions that did not experience an outbreak, but do have suitable conditions such as competent vectors. Conducting vaccine trials in disease outbreaks is complex, but there are tools to facilitate planning [45]. ZIKV in an endemic setting, such as in Africa and Asia, could prove a suitable setting as well. However, ZIKV circulation in endemic setting is not well described and the occurrence of adverse outcomes in this context is less documented [9]. Further research in understanding the transmission of the virus in an endemic context is therefore needed. Similarly, we need to increase the understanding of changes over time in vector abundance and population composition, since these influence the risk of new outbreaks.

### Conclusion

Preparedness is vital; the time until the next outbreak gives us the opportunity to be prepared. The next sizeable ZIKV outbreak in Nicaragua will likely not occur before 2035 but the probability of outbreaks will increase. Young women of reproductive age will be at highest risk of infection during the next ZIKV outbreak. Vaccination targeted to young women could curb the risk of a large outbreak and extend herd immunity. ZIKV vaccine development and licensure are urgent to attain the maximum benefit in reducing the population-level risk of infection and the risk of adverse congenital outcomes. The urgency of ZIKV vaccine development increases if immunity is not lifelong.

## Supporting information

S1 Text Supplementary model description. (PDF)

S2 Text Sensitivity analysis. (PDF)
