# Supplementary material for: Impact of age-specific immunity on the timing and burden of the next Zika virus outbreak
Source: PLoS Negl Trop Dis. 2019 Dec 26;13(12):e0007978. doi: 10.1371/journal.pntd.0007978 (PMC6948816; doi:10.1371/journal.pntd.0007978)
Supplement: S1 Text — (PDF) [file pntd.0007978.s001.pdf]

Supporting information 1. Supplementary model description

Supplement to:

Impact of age-specific immunity on the timing and burden of the next Zika virus outbreak

Michel J. Counotte, Christian L. Althaus, Nicola Low and Julien Riou

Contents

S1.1 Model workflow 2

S1.2 Comparison of SIR model with SEIR model and the Pandey model. 3

S1.3 Prior predictive check 3

S1.4 Comparison of “square root normal”, “negative binomial” and “Poisson” approaches 4

S1.5 $\mathcal{R}_0$ calculation 5

S1.6 Loss of immunity scenarios 5

S1.7 ABM algorithm 6

S1.8 The number of infections introduced does influence the probability of an outbreak, but not the attack rate of successful outbreaks 7

S1.1 Model workflow

Figure 1 provides a workflow of the parameterization using an ODE model and subsection predictions using an ABM model.

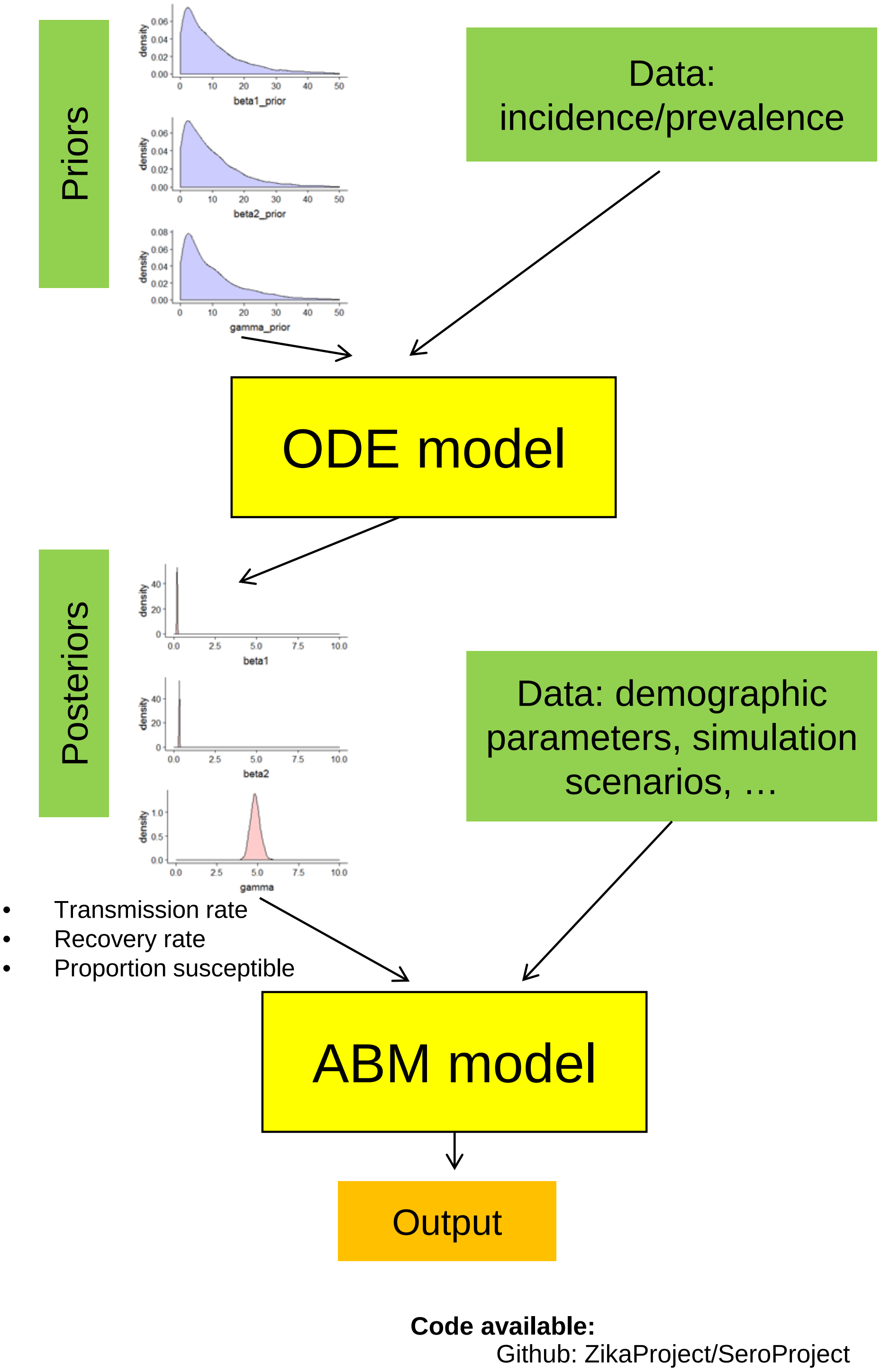

Figure 1: Graphic description of the model.

S1.2 Comparison of SIR model with SEIR model and the Pandey model.

We chose a simple SIR structure to model the transmission of ZIKV (Fig. 2). Other common choices include SEIR structures, including an incubation period, and a Pandey-type structure explicitly modelling the vector population as implemented in Champagne et al. (2016) [1]. We support this choice by conducting model selection using leave-one-out cross-validation (LOO-CV) [2]. The objective of LOO-CV is to estimate the leave-one-out information criterion (LOOIC), a measure of the pointwise out-of-sample prediction accuracy from a fitted Bayesian model. The estimation of the LOOIC relies on Pareto smoothed importance sampling (PSIS), a procedure for regularizing importance weights.

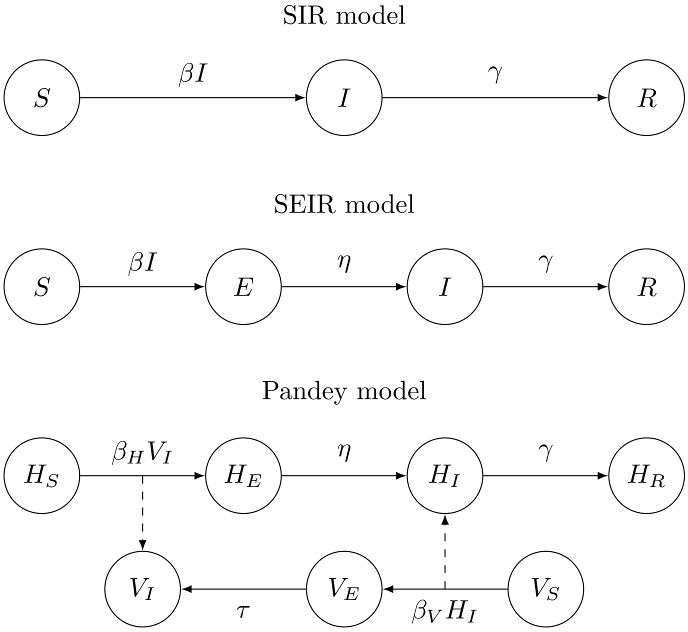

Figure 2: Description of the three compared models. These diagrams ignore the stratification in two age groups: 0-14 and ≥15.

We compared the fits of the SIR, SEIR and Pandey model to incidence and seroprevalence data from Managua, Nicaragua. The LOOIC for the SEIR model (93.9) was slightly lower than for the SIR model (95.2) but the the estimated pointwise difference in LOOIC (ΔLOOIC) of -1.3 was small compared to its standard error, indicating no evidence in support of a better fit. The ΔLOOIC between the SIR and Pandey models was also small and in favour of the SIR model. Overall, this model selection approach supported our choice of the SIR model.

Table 1: Model comparison

Model	LOOIC (SE)	ΔLOOIC (SE)
SIR	95.2 (8.7)	Ref.
SEIR	93.9 (8.9)	-1.3 (1.6)
Pandey	99.5 (8.3)	+4.3 (3.6)

S1.3 Prior predictive check

The choice of prior distribution is a crucial aspect of analyses conducted in a Bayesian framework. Prior predictive checks can be used to assess the adequacy of the choice of prior distributions [3]. The principle is to use the model to simulate artificial data from the chosen set of prior distribution. If the chosen set of priors can lead to any dataset that could plausibly be observed, then the priors can be qualified as "non-informative". Figure 3 shows that this is indeed the case here, as our choice of priors for β_1, β_2, γ, ρ

and $I(0)$ lead to a wide variety of possible epidemic data, from 0 to 4,000 cases reported weekly and from 0 to 100% post-epidemic seroprevalence.

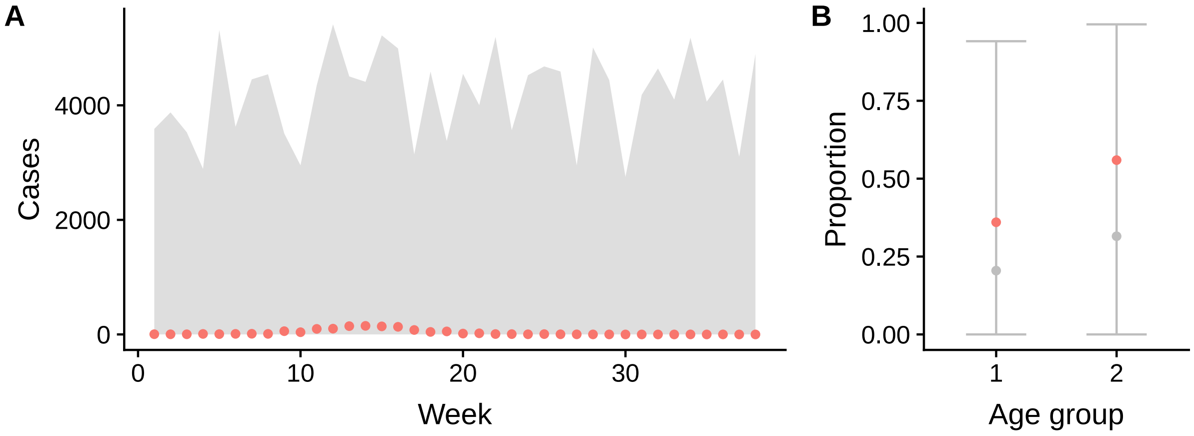

Figure 3: Prior predictive check for the model. (A) Weekly number of reported cases of Zika virus infection in Managua, Nicaragua (grey area shows the 95% range in the artificial data simulated from the prior distributions, red dots show actual data). (B) Post-epidemic seroprevalence (grey bar shows the 95% range in the artificial data, red dots are actual data).

S1.4 Comparison of "square root normal", "negative binomial" and "Poisson" approaches

Several approaches can be used to fit an ODE model to incidence data. The objective is to obtain the joint posterior distribution of the parameters β_1, β_2 and γ by considering the likelihood of the incidence data A. A straightforward choice for modelling count data is to use a Poisson distribution to link the output of the ODE system at time t D_t to weekly incidence data A_t:

$$\Pr(A|\beta_1, \beta_2, \gamma) = \prod_t \text{Poisson}(A_t|C_t) \tag{1}$$

A common problem using Poisson distributions is the presence of overdispersion. A direct solution is to use instead a negative binomial distribution, with an additional overdispersion parameter ϕ:

$$\Pr(A|\beta_1, \beta_2, \gamma) = \prod_t \text{Neg-Bin}(A_t|C_t, \phi) \tag{2}$$

However, the classical negative binomial distribution can struggle when data varies from 0 to large values, as it results in variance estimates that do not scale properly. A solution is to use a modified negative binomial distribution where the overdispersion parameter is scaled by the mean, so that:

$$\Pr(A|\beta_1, \beta_2, \gamma) = \prod_t \text{Neg-Bin}(A_t|C_t, C_t \times \phi) \tag{3}$$

An alternative is to use a normal distribution after a square-root transformation aimed at stabilizing the variance, as described in [4]:

$$\Pr(A|\beta_1, \beta_2, \gamma) = \prod_t \mathcal{N}(\sqrt{A_t}|\sqrt{C_t}, \sigma) \tag{4}$$

We decided to use this last solution in our model, but provide here a comparison of the model fit (Figure 4) and parameter estimates (Table 2) obtained with the other approaches. We show that all the approaches lead to very similar fits and parameter estimates, although the Poisson approaches leads to narrower credible intervals.

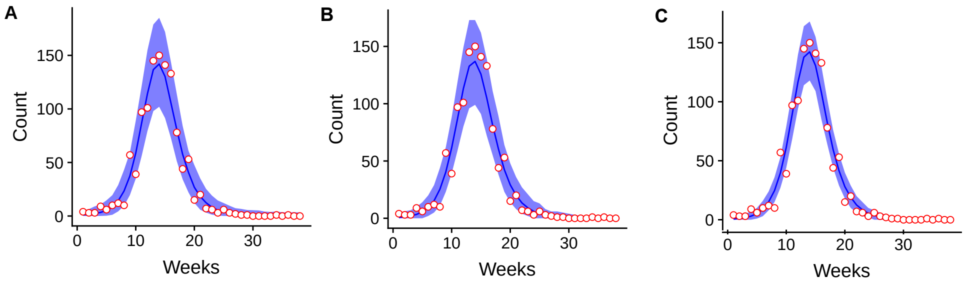

Figure 4: Comparison of fit with Sqrt (A), Poisson (B), and Modified negative binomial (C).

Table 2: Comparison of parameter estimates with Sqrt, negative binomial, and Poisson.

Approach	Square root normal	Poisson	Modified negative binomial
β_1	0.17 (0.15–0.20)	0.16 (0.14–0.18)	0.17 (0.15–0.19)
β_2	0.31 (0.28–0.34)	0.30 (0.27–0.32)	0.31 (0.29–0.33)
$1/\gamma$	5.24 (4.69–5.86)	5.58 (4.98–6.21)	5.35 (4.85–5.84)

S1.5 $\mathcal{R}_0$ calculation

We used the next generation matrix method described by Diekmann et al. (2010) to calculate $\mathcal{R}_0$ (eq. 5 - 7) [5]. β_1 is the transmission rate for the 0–14 age group; β_2 for the >15 group and γ is the common recovery rate.

We start by expressing the model with the infection matrix F and the migration matrix V:

$$F = \begin{pmatrix} \beta_1 & \beta_1 \\ \beta_2 & \beta_2 \end{pmatrix} \tag{5}$$

$$V = \begin{pmatrix} -\gamma & 0 \\ 0 & -\gamma \end{pmatrix} \tag{6}$$

$\mathcal{R}_0$ is defined as the square root of the largest eigenvalue of FV^{-1}:

$$\mathcal{R}_0 = \sqrt{\frac{\beta_1 + \beta_2}{\gamma}} \tag{7}$$

S1.6 Loss of immunity scenarios

We explored plausible scenarios of loss of immunity with mean durations of 15, 30, 60, 90, and 150 years (Fig. 5).

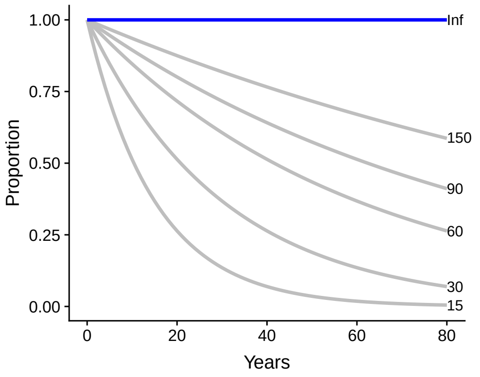

Figure 5: Different scenarios considered regarding the loss of immunity.

S1.7 ABM algorithm

Here, we provide pseudo code of the ABM (Algorithm 1).

Algorithm 1 ABM

```
procedure Initialization                  ▷ Add initial conditions S/R and sex per n individual
    for n ← 1, popMax do
        R[n] ← select random 1 or 0 with probability(age[n])
        S[n] ← 1 − R[n]
        I[n] ← 0
        sex[n] ← select random 1 or 0 with probability 0.5
    end for
end procedure
procedure Simulation                      ▷ Simulation over wkMax weeks
    for wk ← 1, wkMax do
        for n ← 1, popMax do              ▷ Loop over popMax individuals
            if individual is alive then
                procedure Population dynamics          ▷ Pre-outbreak
                    Birth, Death, Ageing
                end procedure
                procedure Loss of Immunity             ▷ Loss of immunity
                    [R → S] with probability RateToProb(ξ)
                end procedure
                procedure Vaccination                  ▷ Vaccination
                    [S → R] with probability vaccinationProb, at age[n]
                end procedure
                procedure Infection, Recovery          ▷ During outbreak
                    [S → I] with probability RateToProb(β, age[n])
                    [I → R] with probability RateToProb(γ)
                end procedure
            end if
        end for
        procedure Start outbreak                       ▷ Introduction of infection
            if wk = introductionWk then
                Change timestep: 7 days to 0.1 days
                Collect summary statistics pre-outbreak
                Introduce introductionN infections
            end if
        end procedure
        total number alive                             ▷ Collect summary of week wk:
        total number infected
    end for
end procedure
```

S1.8 The number of infections introduced does influence the probability of an outbreak, but not the attack rate of successful outbreaks

The proportion of outbreaks (1% threshold) after introduction depends on the number of infections introduced; the attack rate of the successful outbreaks does not depend on the number of infections introduced (Fig. 6).

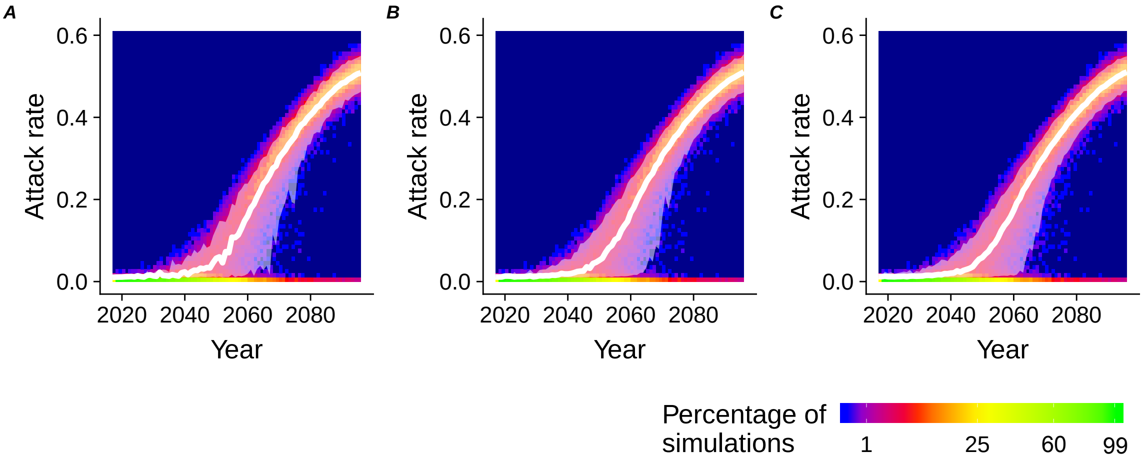

Figure 6: Attack rate over time for the introduction of (A) n=1, (B) n=5, (C) n=10 infections.

References

[1] Champagne C, Salthouse DG, Paul R, Cao-Lormeau VM, Roche B, Cazelles B. Structure in the variability of the basic reproductive number (R0) for Zika epidemics in the Pacific islands. Elife. 2016;5. doi:10.7554/eLife.19874.

[2] Vehtari A, Gelman A, Gabry J. Practical Bayesian model evaluation using leave-one-out cross-validation and WAIC. Statistics and Computing. 2017;27(5):1413–1432. doi:10.1007/s11222-016-9696-4.

[3] Gabry J, Simpson D, Vehtari A, Betancourt M, Gelman A. Visualization in Bayesian workflow. Journal of the Royal Statistical Society: Series A (Statistics in Society). 2019;182(2):389–402. doi:10.1111/rssa.12378.

[4] Guan Y. Variance stabilizing transformations of Poisson, binomial and negative binomial distributions. Stat Probab Lett. 2009;79(14):1621–1629. doi:10.1016/j.spl.2009.04.010.

[5] Diekmann O, Heesterbeek JAP, Roberts MG. The construction of next-generation matrices for compartmental epidemic models. J R Soc Interface. 2010;7(47):873–885. doi:10.1098/rsif.2009.0386.
