# Supplementary material for: Impact of age-specific immunity on the timing and burden of the next Zika virus outbreak
Source: PLoS Negl Trop Dis. 2019 Dec 26;13(12):e0007978. doi: 10.1371/journal.pntd.0007978 (PMC6948816; doi:10.1371/journal.pntd.0007978)
Supplement: S2 Text — (PDF) [file pntd.0007978.s002.pdf]

Supporting information 2. Sensitivity analysis

Supplement to:

Impact of age-specific immunity on the timing and burden of the next Zika virus outbreak

Michel J. Counotte, Christian L. Althaus, Nicola Low and Julien Riou

Contents

S2.1 Introduction 1

S2.2 Seasonality 1

S2.3 Varying vector densities 3

S2.4 Migration 3

S2.5 Endemic transmission 4

References 6

S2.1 Introduction

The results regarding the future risk of ZIKV outbreak in Managua, Nicaragua presented in the main analysis rely upon several hypotheses and modelling choices. The potential effects of two main points of uncertainty, the rate of immunity loss and the introduction of targeted vaccination, were evaluated in the main text. Here, we assess the potential effects on our results of several additional features that were not considered in the main analysis.

S2.2 Seasonality

Variations in vector abundance according to the season may result in a variation in the transmission rate according to yearly cycles. We explored the effect of seasonality using the approached proposed by Netto et al. (2017) [1], that is based on a forcing cosine function f with a frequency of 52 weeks (equation 1). The amplitude α and the shift κ of the cosine function are estimated from data:

$$f(t,\alpha,\kappa) = 1 + \alpha \times \cos\left(\frac{6.283(t-\kappa)}{52}\right) \quad (1)$$

All things being equal, introducing seasonality will lead to an increase of the estimated transmission rate at certain times of the year, as in this case the decrease in incidence towards the end of the epidemic is not only caused by a lack of susceptibles, but also by the seasonal decrease in transmission. In forward simulations, this may lead to an earlier increase of the risk of outbreak if introductions happen at a favourable time. However, as the shift of the seasonal cycle κ is estimated from data, this model assumes that disease introduction in the population took place on the most optimal time. This could lead to an underestimation of the transmission rate if disease introduction occurred at a less optimal time in the season.

We compare our baseline model ignoring seasonality ("No seasonality", Fig. 1A-D) with a model including seasonal forcing with α and κ informed by data ("Flexible seasonal forcing", Fig. 1E-H) and

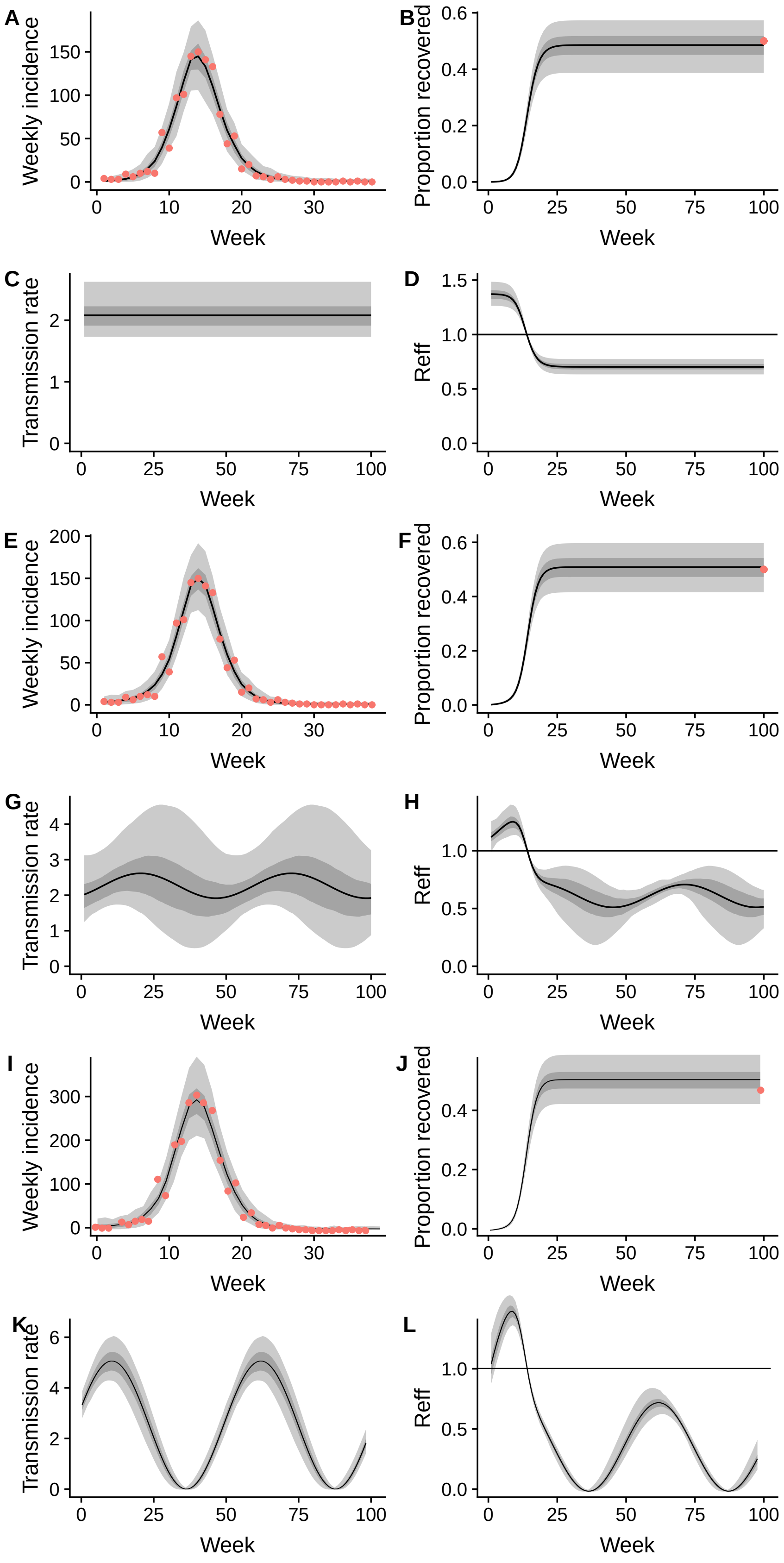

Figure 1: Model implemented without seasonality (A-D). Model implemented with flexible seasonal forcing: κ and α are estimated from data (E-H). Model implemented with full seasonal forcing: κ is estimated from data and α is fixed at 1 (I-L).

a model including seasonal forcing with α fixed to 1 ("Full seasonal forcing", Fig. 1I-L). Compared to no seasonality, including a flexible seasonal forcing leads to a peak estimate of $\mathcal{R}_0$ higher by 6%, and including full seasonal forcing higher by 12%.

We considered the impact of an augmentation of the transmission rate by 12% on the model predictions (Fig. 3, scenario n°2). Such an increase would result in a reduction of the time window before observing a rise in the risk of ZIKV outbreak (21 years until 50% of reintroductions result in outbreaks, compared with 31 years in the baseline model) and an increase of the attack rate at year 2047 to 15.3% (IQR: 11.2–18.4), compared with 3.6% (IQR: 2.0–6.2) in the baseline model.

S2.3 Varying vector densities

Changes in vector density may result in an increase or a decrease of the transmission rate. Vector densities may change over time according to human population densities and climate [2]. The complex interactions between climate, human demography and vector abundance make long term predictions of future vector abundance difficult. Therefore, we considered the impact of both an increase and a decrease of the transmission rate on our estimate of the future risk of ZIKV outbreak.

As reported in the previous section, an increase of the transmission rate by 12% would result in an earlier increase of the risk of outbreak and higher average attack rates (Fig. 3, scenario n°2). Conversely, a decrease of the transmission rate by 12% would extend the time window before observing a rise in the risk of outbreak (44 years until 50% of the simulated introductions result in outbreaks, Fig. 3, scenario n°3). The average attack rate in 2047 would be 1.6% (IQR: 1.3–2.5).

S2.4 Migration

Human migration may impact the future evolution of the proportion of susceptible individuals in Managua, Nicaragua. We considered the effect of urbanization, or an influx of rural inhabitants, which is a plausible scenario in this particular context [3]. Evidence suggests that rural populations have lower seroprevalence [4]. Urbanization might thus result in a quicker decline of protective immunity in the population than expected.

The International Institute for Applied Systems Analysis (IIASA) produces predictions of population and urbanization according to different Shared Socioeconomic Pathways (SSP) storylines. SSP storylines are different narrative scenarios of how trends change over time [5]. We considered a scenario where the projected urbanization in Nicaragua follows the SSP2 or "middle of the road" storyline [6]. This implies that the proportion of urbanization in Nicaragua will rise from 60 to 79% between 2015 and 2100 (Fig. 2). We considered an extreme situation where urbanization consists of an influx of fully susceptible individuals with a median age of 30. Under these conditions, the time until 50% of reintroductions result in an outbreak would decrease from 31 to 23 years and the attack rate in 2047 is 12.7% (IQR: 8.7–16.2) (Fig. 3, scenario n°1).

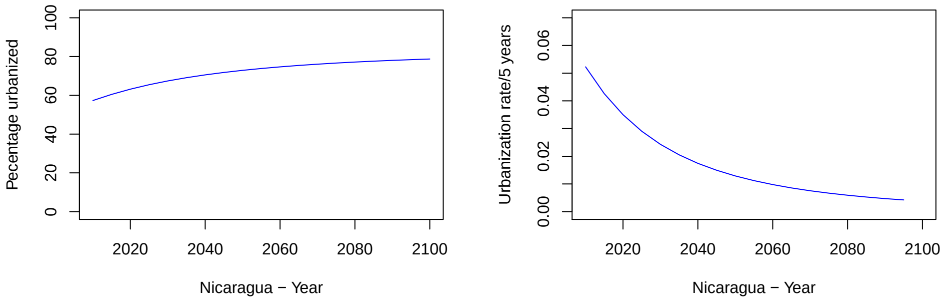

Figure 2: Proportion and rate of urbanization in Nicaragua according to the SSP2 scenario [6].

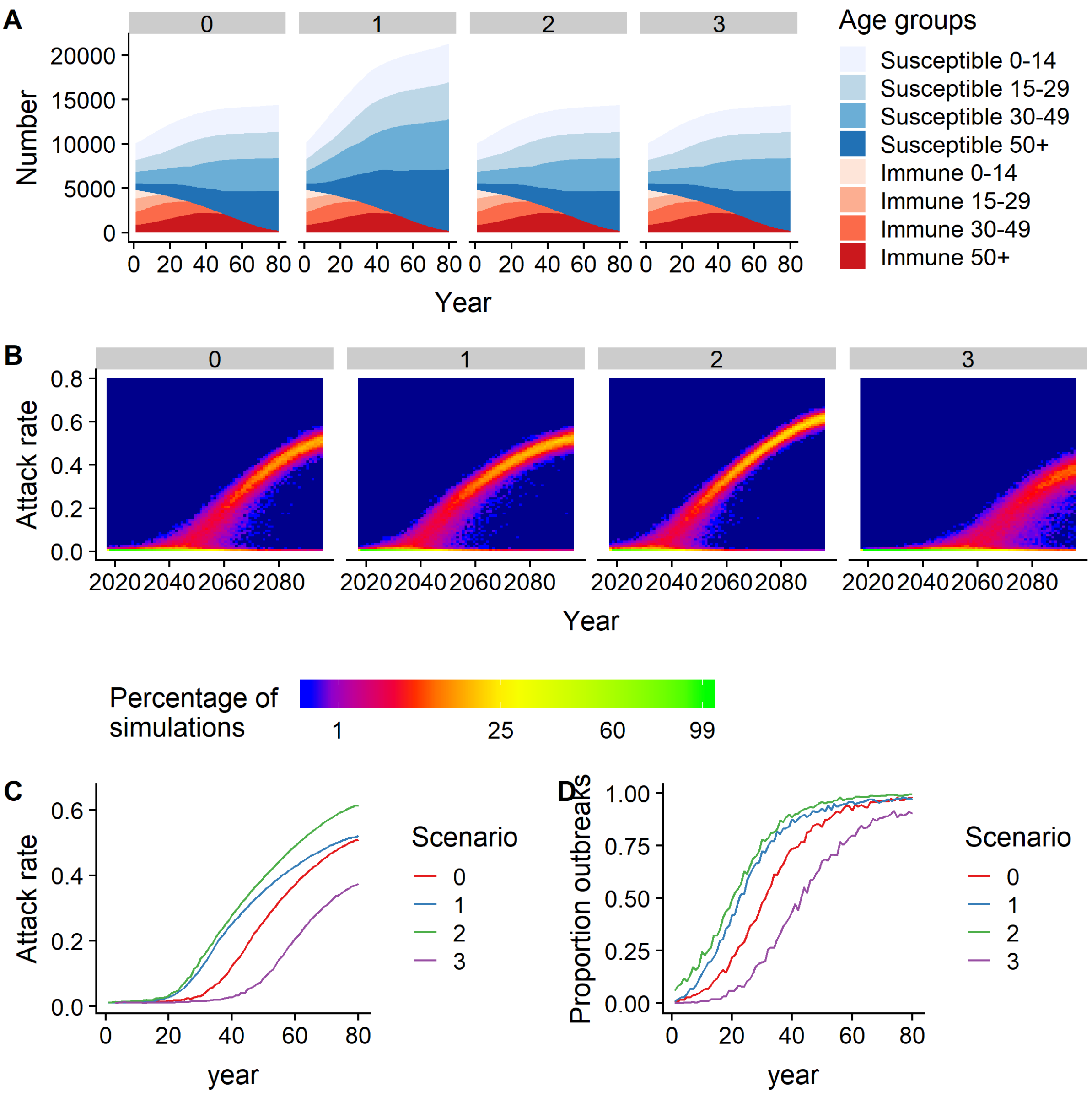

Figure 3: Comparison of the predicted evolution of protective immunity (A); the distribution (B) and the average (C) of the attack rates resulting from the reintroduction of ZIKV in the population each year; and the proportion of reintroductions resulting in an outbreak with attack rate ¿1% across four modelling scenarios: baseline scenario used in the main analysis (n°0), scenario including migration from rural areas (n°1), scenario corresponding to a transmission rate increased by 12% (n°2) or decreased by 12% (n°3).

S2.5 Endemic transmission

In the main analysis, we considered that ZIKV entirely disappeared from Managua, Nicaragua after the epidemic waves of 2015-2017. This assumption is coherent with the sharp decline in reported cases of ZIKV on the continent after 2017 and the limited evidence on ZIKV infection of new world monkeys (limiting the establishment of sylvatic endemic circulation cycles [7]). If low level circulation exists and ZIKV becomes endemic in the Americas, repeated reintroductions in Managua, Nicaragua would occur which would have implications on our projections.

We considered this scenario by modifying the rate of reintroduction of ZIKV in our simulations. In the main model, we consider a single introduction per simulation, corresponding to an epidemic setting where ZIKV does not circulate and has to be reintroduced from outside. To mimic endemic circulation, we consider continuous reintroductions of ZIKV into our population with a monthly probability of 1/12

(on average one introduction per year), in the presence and absence of seasonality (Fig. 5).

With these conditions, the time until 50% of the simulations result in an outbreak where at least 1% of the population is affected would be 23 years in absence of seasonality, and 22 years when seasonality was considered (Fig. 4). The median attack rate in 2047 would be 2.5% (IQR: 1.7–4.1) in the absence of seasonality and 5.1% (IQR: 2.3–13.9) when seasonality is considered. Continuous reintroductions would also result in a lot more variability due to stochasticity.

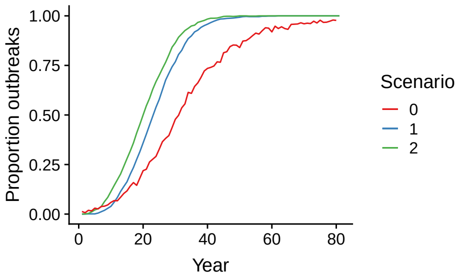

Figure 4: Comparison of the different ABM simulation scenarios taking into different levels of endemicity and seasonality and the effect on the proportion of outbreaks. Baseline scenario with one reintroduction per simulation (0), continuous reintroductions without seasonality (1), continuous reintroductions with seasonality (2).

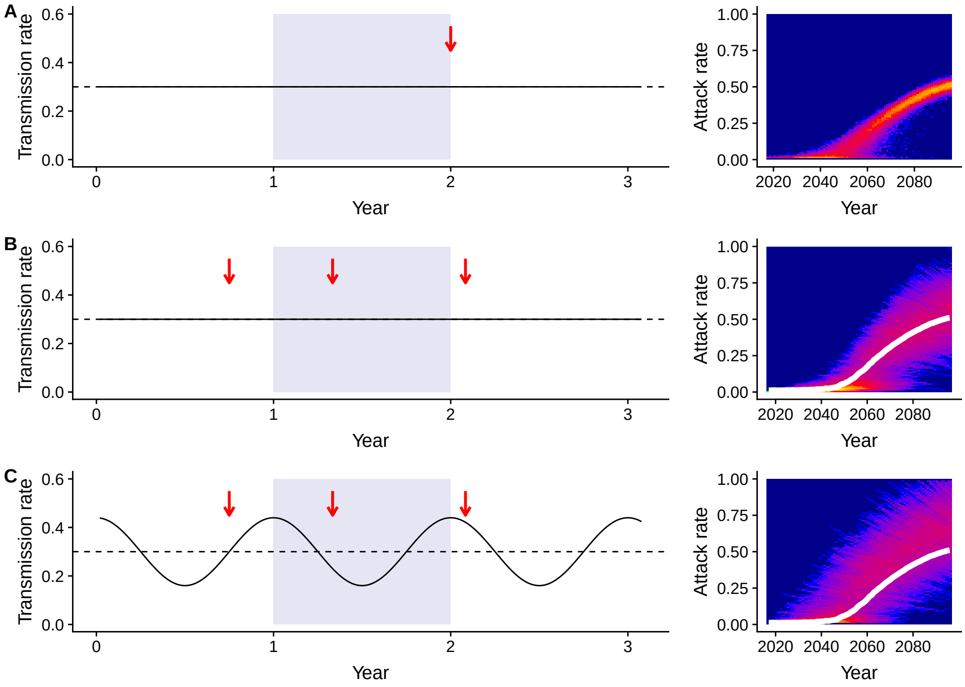

Figure 5: Comparison of the different ABM simulation scenarios taking into different levels of endemicity and seasonality. Red arrows represent ZIKV reintroductions. (A) A single reintroduction per simulation with no seasonal fluctuation of the transmission rate, baseline scenario. (B) Random continuous reintroductions each year without seasonality. (C) Random continuous reintroductions each year with seasonality. The thick white line in panel B and C, represent the median attack rate of the baseline model (A) for comparison.

References

[1] Netto EM, Moreira-Soto A, Pedroso C, Höser C, Funk S, Kucharski AJ, et al. High Zika Virus Seroprevalence in Salvador, Northeastern Brazil Limits the Potential for Further Outbreaks. MBio. 2017;8(6). doi:10.1128/mbio.01390-17.

[2] Kraemer MUG, Reiner RC, Brady OJ, Messina JP, Gilbert M, Pigott DM, et al. Past and future spread of the arbovirus vectors Aedes aegypti and Aedes albopictus. Nat Microbiol. 2019;4(5):854–863. doi:10.1038/s41564-019-0376-y.

[3] Maria A, Acero JL, Aguilera AI, Lozano MG. Central America Urbanization Review: Making Cities Work for Central America. The World Bank; 2017.

[4] Langerak T, Brinkman T, Mumtaz N, Arron G, Hermelijn S, Baldewsingh G, et al. Zika Virus Seroprevalence in Urban and Rural Areas of Suriname, 2017. J Infect Dis. 2019;doi:10.1093/infdis/jiz063.

[5] O'Neill BC, Kriegler E, Ebi KL, Kemp-Benedict E, Riahi K, Rothman DS, et al. The roads ahead: Narratives for shared socioeconomic pathways describing world futures in the 21st century. Global Environmental Change. 2017;42:169 – 180. doi:10.1016/j.gloenvcha.2015.01.004.

[6] International Institute for Applied Systems Analysis . SSP Database (Shared Socioeconomic Pathways) - Version 2.0; 2019. [Online]. Available from: https://secure.iiasa.ac.at/web-apps/ene/SspDb/dsd?Action=htmlpage&page=citation.

[7] Althouse BM, Vasilakis N, Sall AA, Diallo M, Weaver SC, Hanley KA. Potential for Zika Virus to Establish a Sylvatic Transmission Cycle in the Americas. PLOS Neglected Tropical Diseases. 2016;10(12):1–11. doi:10.1371/journal.pntd.0005055.
